# Just Drink a Glass of Water? Effects of Bicarbonate–Sulfate–Calcium–Magnesium Water on the Gut–Liver Axis

**DOI:** 10.3389/fphar.2022.869446

**Published:** 2022-06-28

**Authors:** Antonietta Gerarda Gravina, Mario Romeo, Raffaele Pellegrino, Concetta Tuccillo, Alessandro Federico, Carmelina Loguercio

**Affiliations:** Department of Precision Medicine, University of Campania “Luigi Vanvitelli”, Naples, Italy

**Keywords:** mineral water, gastrointestinal hormones, oxidative stress, gut microbiota, NAFLD

## Abstract

**Background and Aim:** Fonte Essenziale® water is a bicarbonate–sulfate–calcium–magnesium water, low in sodium, recognized by the Italian health care system in hydropinotherapy and hepatobiliary dyspepsia therapy. We wanted to explore its effects on the gut–liver axis and microbiota in non-alcoholic fatty liver disease patients.

**Patients and Methods:** We considered enrollment for 70 patients, of which four were excluded. We finally enrolled 55 patients with ultrasound-documented steatosis (SPs+) and 11 patients without it (SPs−). They then drank 400 ml of water for 6 months in the morning on an empty stomach. Routine hematochemical and metabolic parameters, oxidative stress parameters, gastrointestinal hormone levels, and fecal parameters of the gut microbiota were evaluated at three different assessment times, at baseline (T0), after 6 months (T6), and after a further 6 months of water washout (T12). We lost, in follow-up, 4 (T6) and 22 (T12) patients.

**Results:** Between T0–T6, we observed a significant Futuin A and Selenoprotein A decrease and a GLP-1 and PYY increase in SPs+ and the same for Futuin A and GLP-1 in SPs−. Effects were lost at T12. In SPs+, between T0–T12 and T6–12, a significant reduction in Blautia was observed; between T0–T12, a reduction of Collinsella unc. was observed; and between T0–T12 and T6–12, an increase in Subdoligranulum and Dorea was observed. None of the bacterial strains we analyzed varied significantly in the SPs− population.

**Conclusion:** These results indicate beneficial effects of water on gastrointestinal hormones and hence on the gut–liver axis in the period in which subjects drank water both in SPs− and in SPs+.

## Introduction

Non-alcoholic fatty liver disease (NAFLD) is the main cause of chronic hepatopathy in Western regions. The diagnosis of NAFLD requires the detection, generally performed with a non-invasive radiological technique, of an intrahepatic fatty accumulation in more than 5% of hepatocytes after having excluded other causes of chronic hepatopathy (e.g., significant alcohol consumption, viral forms, autoimmunity, or iatrogenic forms) (Byrne and Targher, 2015). However, a recent consensus has stigmatized the use of a new definition, namely, that of metabolic (dysfunction) associated fatty liver disease (MAFLD), which incorporates the metabolic phenotype of NAFLD but does not rigidly exclude alcohol consumption in its definition (Eslam et al., 2020). Currently, NAFLD showed a prevalence ranging from 15% to 25% of the general population in various countries, with values ranging from 20% to 40% in the United States and 10%–20% in Asian countries (McCullough, 2004). In Italy, NAFLD has a prevalence of approximately 25% (Shifflet and Wu, 2009) with an increase of up to 70%–90% in obese patients or patients with type 2 diabetes mellitus (Neuschwander-Tetri, 2005; Caserta et al., 2017). Moreover, in Italy, there has been a significant increase, in etiological terms, over time of metabolic liver disorders, and hence NAFLD, as early as the first decade of 2000 (Saracco et al., 2016).

Several pathophysiological mechanisms have been implicated in the pathogenesis of NAFLD, including alterations in the gut microbiota and gut–liver axis, with changes in intestinal permeability and intestinal inflammation with the migration of microbial pathogen-associated molecular patterns (PAMPs) *via* the portal circulation to the liver (Lau and Wong, 2018; Abenavoli et al., 2021). In the NAFLD patient’s gut microenvironment, several alterations in microbial composition occur, often with the formation of different microbiota “signatures” (Aron-Wisnewsky et al., 2020). In general, some microbial populations (e.g., Bacteroidetes, Ruminococcus, Dorea, Blautia, and Firmicutes) seem to be subject to significant variations (Federico et al., 2016a, 2016b; Patrone et al., 2016; Lau and Wong, 2018). Alterations of the gut–liver axis have, moreover, been repeatedly called into question in NAFLD pathogenesis (Abenavoli et al., 2021). This axis is based on the whole series of interactions that the gut has with the liver thanks to signals triggered by genetic, dietary, and environmental factors, and this is established by the vascular network provided by the portal system (Albillos et al., 2020).

In addition, the gut–liver axis plays a key role in regulating the “trafficking” of gut-derived substances (i.e., PAMPs), which may lead to liver damage. Moreover, the increase of gut permeability already identified in NAFLD, according to the theory of “leaky gut syndrome,” favors the passage of endotoxins from the gut to the liver through the portal circulation damaging it. Endotoxins from the intestine could activate the Kupffer cells, inducing an increase in the production of pro-inflammatory cytokines, such as IL-6, IL-12, IL-18, and TNF-alpha (Cariello et al., 2010; Simbrunner et al., 2019; Di Ciaula et al., 2020). Part of this complex axis is several mediators and enterokines such as glucagon-like peptide 1 (GLP-1), selenoprotein P (SeP), human glycoprotein Fetuin-A (aHSGF), and YY peptide (PYY) (Federico et al., 2016a, 2016b; Patrone et al., 2016; Petta et al., 2016; Meex and Watt, 2017; Sumida and Yoneda, 2018). In detail, it was seen how the levels of these mediators varied in NAFLD with SeP (Chen et al., 2021) and aHSGF (Pan et al., 2020) appearing to be increased and PYY and GLP-1 levels reduced (Tarantino and Balsano, 2020). Last, another mainstay in the pathogenesis of NAFLD is certainly oxidative stress (Ore and Akinloye, 2019; Dallio et al., 2021). Excessive production of reactive oxygen species results in toxic oxidative stress to the liver. It has been hypothesized that such oxidative overload may find its way into NAFLD through the dysregulation of several nuclear receptors involved in regulating the expression of several hepatic lipid homeostasis genes (Hong et al., 2021).

The therapeutic strategy of NAFLD is also composed of non-pharmacological therapeutic interventions (caloric restriction, increase in physical activity, elimination of alcoholic and fructose-rich beverages) (European Association for the Study of the Liver, 2016); however, it has not been well explored whether changing the quality of mineral water normally drunk by patients could have a beneficial role if not for a few pre-clinical and clinical experiences on hydrogen-rich waters (Nakao et al., 2010; Korovljev et al., 2019). Among the drinking waters sold in Italy, Fonte Essenziale® is mineral water that flows from the Boario Thermal Baths, in Valle Camonica, at the foot of Monte Altissimo. This water is bicarbonate–sulfate–calcium–magnesium and low in sodium. The Italian national health system recognized the role of Fonte Essenziale® in hydropinotherapy and, in particular, in the treatment of hepato-biliary dyspepsia (Indicazioni per le etichette dell’acqua minerale «Antica Fonte», in comune di Darfo Boario Terme) (15A00431) (GU Serie Generale n, 2015). Initial work has shown several positive properties of this water on the hepatobiliary system by increasing cholecystokinin and, consequently, increasing gallbladder contractility (Coiro et al., 2002). Cholecystokinin causes an increase in the contraction and emptying of the gallbladder and a reduction in its volume after the prandial stimulus (Byrnes et al., 1981). Similar beneficial effects have also been observed, in terms of oxidative stress, in clinical models of chronic alcoholism, in increasing glutathione levels (Coiro and Vescovi, 1995). To date, there is not enough evidence on what impact Fonte Essenziale® water may have on non-invasive serological markers of liver functions, on the levels of the main mediators of the gut–brain axis, and on the microenvironment of the gut microbiota in patients with NAFLD.

This study aims to evaluate whether Fonte Essenziale® water can impact routine hematochemical parameters, on GLP-1, SeP, PYY, and aHSGF levels as well as on different bacterial species of gut microbiota in a sample of patients diagnosed with NAFLD compared to a liver disease-free population.

## Patients and Methods

### Study Design and Setting

The study design is set up as a prospective longitudinal interventional study. The study was conducted at the Hepatogastroenterology Unit of the University of Campania “Luigi Vanvitelli.” Patient enrollment took place from September 2018 to September 2019. All patients signed an informative consensus before being enrolled, and this study has received approval from the ethics committee of the University of Study of Campania “L. Vanvitelli” (protocol number 682, 27 November 2017). The study protocol conforms to the ethical guidelines of the 1975 Declaration of Helsinki. All enrolled patients drank Fonte Essenziale® water for 6 months at a dosage of 400 ml in the morning on an empty stomach. They underwent, at the time of the first visit (T0), a blood sample, abdominal ultrasound, and delivered a stool sample. After 6 months from the start of water consumption, the patients were recalled for a follow-up visit (T6) and they repeated an abdominal ultrasound and a blood sample, and they again gave us a fecal sample. Patients were asked to return for a follow-up after 6 months after the suspension of water intake (T12). At 12 months, they underwent the same tests of T0. For evaluating the compliance with water intake, we used (T6) a questionnaire that assessed the satisfaction of the subjects with the water intake and their constancy in the intake; however, during the drinking period, we phoned the patients to find out if they were continuing to drink the water and if it was to their liking. For all patients, we collected clinic-demographic variables [sex, age, and body mass index (BMI)]. Homeostatic model assessment (HOMA) index has been calculated at each time of study evaluation (which is the ratio of the product of blood glucose to fasting insulin divided by 22.5 considering expressing blood glucose in mmol/L and insulin in mU/L) (Matthews et al., 1985).

### Inclusion and Exclusion Criteria

Enrolled patients had to meet the following inclusion criteria: patients who came to our outpatient unit to undergo a gastroenterological or hepatological examination for the detection of gastrointestinal symptoms or the ultrasound finding of liver steatosis plus gastrointestinal symptoms. Regarding patients with liver steatosis, the diagnosis of NAFLD was performed according to current guidelines, that is, excluding the consumption of 30 g or more of alcohol daily for males and 20 g or more for females (European Association for the Study of the Liver, 2016). Patients who had not undergone ultrasonography underwent the same to exclude the diagnosis of steatosis. Enrolled patients underwent a thorough gastroenterological examination to exclude that the symptoms were harbingers of red flags suspicious of organic disease (Hunt et al., 2017; Moayyedi et al., 2017; Serra et al., 2017; Wilkins et al., 2018). Gastrointestinal symptoms were then studied along with the history according to the ROMA IV criteria to validate patients as having functional gastrointestinal disorder (Schmulson and Drossman, 2017).

The following exclusion criteria were also considered: pregnant women, patients under the age of 18 years; patients with additional causes of chronic liver disease (HCV/HBV infection, congenital liver disease, alcohol use, and metal accumulation diseases); patients with renal insufficiency, heart failure, thyroid, and adrenal problems; and evidence of neoplasia. Patients with known psychiatric disorders were similarly excluded. We also excluded patients who did not sign the written informed consent. Patients who met the diagnosis of NAFLD and did not possess the exclusion criteria outlined before were enrolled in the “steatosis positive patients” (SPs+) group. In contrast, in the absence of NAFLD diagnosis, they were enrolled in “steatosis negative patients” (SPs−), and therefore, patients with functional gastrointestinal disorders according to ROME IV were included in this group.

### Evaluation of Blood Parameters

The following routine laboratory parameters were collected at each time of study evaluation (i.e., at T0, T6, and T12): alanine aminotransferase (ALT) and aspartate aminotransferase (AST), gamma-glutamyl transpeptidase (γ-GT), triglycerides, cholesterol, and glycemia according to routine internationally assessed methods (Bobinski and Przedlacki, 1962; Smith and Taylor, 1973; Fossati and Prencipe, 1982).

In addition to these, levels of oxidative metabolites as well as gastrointestinal hormones, were assayed. In detail, the blood samples from patients in each group performed before and after treatment were centrifuged and the serum was frozen at −20°C until use. We evaluated total antioxidant power and thiobarbituric acid reactive substances (TAC, TBARs Cell Biolabs Inc.), total thiols (Cell Biolabs Inc.), glutathione (GSH, Cloud-Clone Corp., United States), GLP-1 (Abbexa, Cambridge, United Kingdom), SeP (Cloud-Clone Corp., United States), aHSGF (Cloud-CloneCorp., United States), and peptide YY (Cloud-Clone Corp., United States) at T0, T6, and T12 in all patients. The protocols provided by the kits’ data sheet were adhered to when performing the laboratory measurements.

### Ultrasound Evaluation

To divide the patients into SPs+ and SPs− groups, as already stated, hepatic ultrasonography was used. In particular, the presence of hepatic steatosis was defined according to Ferraioli and Soares Monteiro (2019). Relative to the degree of steatosis, we defined a liver with mild steatosis when there was a discrepancy in brightness between the liver and right kidney without posterior attenuation, moderate steatosis when there was posterior attenuation in addition to the picture of mild steatosis, and severe steatosis if there was a marked attenuation of the ultrasound beam such that the portal bifurcation was not recognizable (Idilman et al., 2016). The ultrasound evaluation was always made by the same operator, with extensive personal ultrasound clinical casuistry for liver diseases, in order not to have inter-operator variability.

### Microbiota Evaluation

For the evaluation of gut microbiota, 200 mg of feces was collected for each patient and 32,000 bacterial species were analyzed. The unit of measurement used was bacteria/200 mg of feces. Total genomic DNA extracted from 200 mg of stool was captured on a silica membrane in the spin-column format with a QIAamp PowerFecal Pro DNA Kit. The NGS microbiome was performed with QIAseq 16S/ITS panels using a two-step polymerase chain reaction (PCR) workflow for targeted enrichment of 16S and ITS genes. The first step of the PCR incorporated a pool of phased primers to enrich the conserved regions of the 16S gene and the ITS gene. After a reaction cleanup with the QIAseq beads, library amplification introduces sample indices and ensures that a sufficient target for NGS is present. After a final cleaning, the libraries are quality-checked (Bioanalyzer®) and quantized using a QIAseq Library Quant System. The number of multiplexed samples depends on the number of variable regions analyzed. QIAseq 16S /ITS Smart Control monitors both proper library construction and contamination introduced by the environment or by the user. After sequencing, any introduced environmental contamination can be identified after bacterial/fungal classification. For data analysis, sequencing was performed on an Illumina MiSeq NGS system using a v2 kit with 251 × 2 coupled limit switches or a v3 kit with 276 × 2 coupled limit switches.

### Statistical Analysis

All continuous variables are expressed as mean ± standard derivation (SD), and categorical variables were described as frequencies and percentages (%).

A Kolgoromov–Smirnov test for normality was performed to evaluate if the parametric or non-parametric analysis should be applied. Wilcoxon signed ranks test, t-student test for dependent groups, and t-student for independent groups or Kruskal–Wallis test walls were performed to compare continuous variables among the different groups considering normal and non-normal distribution. Statistical significance was defined in the case of *p*-value < 0.05. Statistical analyses were performed using GraphPad (PRISM) 8 for MAC-OS.

## Results

**FIGURE 1:**
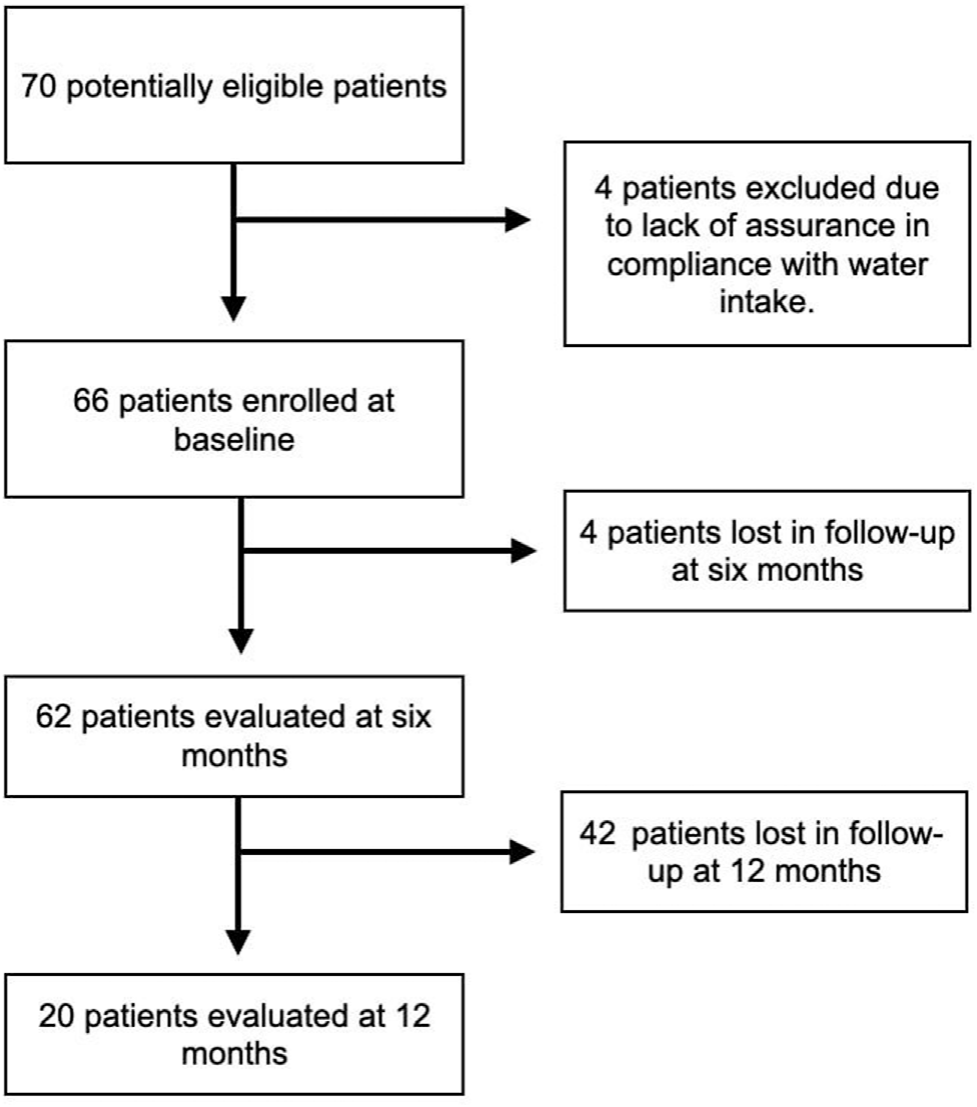
Flowchart summarizing the different phases of patients’ recruitment and follow-up.

A total of 70 patients were considered for enrollment, but four of them were excluded because they could not guarantee a constant consumption of Fonte Essenziale® water as per the study protocol. The main reason given by the four patients was that they did not have a fixed abode as they traveled a lot for work, and this could affect their constant water consumption. During the study, the coronavirus disease 19 (COVID-19) pandemic occurred. Therefore, we lost four patients to follow-up at T6, who were afraid to go to the hospital for fear of becoming infected by COVID-19. Considering the 66 patients enrolled at T0, 20 accepted the invitation to return for a visit at T12, and of these, ten delivered fecal samples. The main reason for this phenomenon was, again, the COVID-19 pandemic. Figure 1 shows the flow chart summarizing the different phases of enrollment and follow-up. Of the 66 patients enrolled, 31 (46.96%) were male, while 35 (53%) were female, and the overall median age was 56.1 years.

On ultrasound evaluation, 55 (83.3%) patients presented evidence of liver steatosis (SPs+), while 11 (16.6%) did not (SPs−). Of the SPs+ patients, 20 (36.36%) had an ultrasound picture of mild steatosis, 30 (54.54%) of moderate steatosis, and finally five (9.09%) of severe steatosis. After 6 months (T6), there was no change in these percentages.

All patients drank the water delivered to them 95% of the time at the recommended dose, and all expressed pleasure in drinking it. Tables 1, 2 show the characteristics related to the variables analyzed at baseline providing the significant differences between the SPs+ and SPs− groups.

**TABLE 1:** Evaluation of study parameters assessed at baseline (T0), at 6 months (T6), and at 12 months (T12) in both patients with (SPs+) and without (SPs−) steatosis.

Parameter	Steatosis-positive patients (SPs+)					Steatosis-negative patients (SPs–)					
	T0	T6	T12	*p*-Value*	*p*-Value**	T0	T6	T12	*p*-Value*	*p*-Value**	*p*-Value†
BMI	29.84 ± 4.218	29.54 ± 4.289	30.21 ± 4.970	0.4221	0.0547	26.52 ± 4.489	25.43 ± 2.994	25.94 ± 3.022	0.8125	0.0625	**0.0285**
AST (UI/l)	23.16 ± 7.396	22.80 ± 6.344	28.56 ± 13.35	0.8815	0.2891	23.36 ± 5.749	19.45 ± 5.429	21.09 ± 4.134	0.0635	0.7549	0.1759
ALT (UI/l)	28.25 ± 15.34	26.10 ± 10.07	29.11 ± 17.22	0.2498	0.5547	29.82 ± 30.17	22.45 ± 10.86	22.36 ± 10.78	0.3975	0.8730	0.3214
γ-GT (U/l)	31.99 ± 26.57	29.71 ± 13.70	26.22 ± 9.692	0.7956	0.3984	24.27 ± 13.20	27.27 ± 14.77	27.73 ± 10.95	0.7793	0.9111	0.1377
Total Cholesterol (mg/dl)	193.3 ± 35.92	197.0 ± 32.88	163.6 ± 59.05	0.3219	0.4180	200.9± 14.56	185.5 ± 41.07	182.8 ± 41.70	0.4258	0.4609	0.3790
Triglycerides (mg/dI)	141.7 ± 61.72	153.1 ± 63.57	116.3 ± 58.34	0.5925	0.691	116.6 ± 28.81	107.5 ± 36.27	113.4 ± 31.18	0.1064	0.3115	0.0893
Glycemia (mg/dl)	101.4 ± 15.54	104.8 ± 20.17	98.44 ± 15.65	0.5825	0.1289	84.27 ± 8.912	85.64 ± 8.140	89 ± 10.49	0.4785	0.2236	**0.0001**
Insulin (μU/ml)	15.09 ± 6.363	13.66 ± 7.690	17.84 ± 9.202	0.1660	0.9102	12.57 ± 6.585	8.708 ± 6.060	9.392 ± 6.104	0.1191	0.5332	0.4089
HOMA-IR	3.712 ± 1.891	3.683 ± 2.367	4.271 ± 1.985	0.6545	0.9688	2.704 ± 1.478	1.903 ±1.502	2.162 ± 2.040	0.1982	0.2402	0.1649
TAC	0.3909 ± 0.8044	0.3006 ± 0.4883	0.2466 ± 0.0822	0.1383	0.1055	0.2501 ± 0.04839	0.2857 ± 0.384	0.5194 ± 0.1425	0.720	**0.0010**	0.1383
TBARs (μM)	1.360 ± 0.8302	1.190 ± 0.5617	0.979 ± 0.4667	0.2153	0.1016	1.297 ± 0.5707	1.373 ± 0.4326	1.355 ± 0.5022	0.4268	0.7002	0.9168
Total thiols (μM)	90.14 ± 76.43	81.24 ± 71.34	43.75 ± 17.48	0.2494	0.7344	90.57 ± 50.26	72.24 ± 51.31	72.83 ± 51.76	0.3652	0.1016	0.5094
GSH (mg/ml)	59.41 ± 57.28	70.21 ± 81.59	39.75 ± 28.85	0.4376	0.1289	63.49 ± 70.48	82.91 ± 74.16	81.63 ± 75.27	0.2695	0.5771	0.8025
aHSGF (ng/ml)	9.752 ± 3.243	7.421 ± 2.494	11.16 ± 2.538	**<0.0001**	**0.0117**	9.537 ± 2.504	7.800 ± 1.706	8.483 ± 1.954	**0.0322**	**0.0029**	0.8296
GLP-1 (pg/ml)	4.279 ± 0.2771	11.137 ± 3.911	7.727 ± 2.613	**<0.0001**	**0.0375**	8.281 ± 3.723	11.92 ± 3.556	4.405 ± 2.263	**0.0420**	**0.0313**	**0.0020**
PYY (pg/ml)	45.50 ± 56.61	98.50 ± 123.1	72.52 ± 105.4	**< 0.0001**	**0.0391**	58.43 ± 64.88	45.71 ± 21.03	46.12 ± 22.33	0.9658	0.4648	0.2962
SeP (ng/ml)	5.918 ± 13.53	1.649 ± 1.862	4.017 ± 3.412	**< 0.0001**	**0.0078**	1.116 ± 0.5851	1.332 ± 1.224	1.453 ± 1.302	0.7646	0.2402	**0.004**

*The *p*-value evaluates whether the change in parameter values between T0–T6 is statistically significant. **The *p*-value evaluates whether the change in parameter values between T6–T12 is statistically significant. ^†^The *p*-value evaluates whether the differences in parameter values between groups (SPs + vs. SPs−) are statistically significant at baseline (T0). The data are expressed as mean ± SD. Significant *p*-values are indicated in bold.

**TABLE 2:** Evaluation of gut microbiota at baseline (T0) and after 6 months (T6) for patients with (SPs+) and without steatosis (SPs−) and also at 12 months (T12) for patients with steatosis.

Bacterial strain considered	Steatosis-positive patients (SPs+)						Steatosis-negative patients (SPs–)			
	T0	T6	T12	*p*-Value*	*p*-Value**	*p*-Value***	T0	T6	*p*-Value*	*p*-Value†
Bacteria, Actinobacteria, Bifidobacteriaceae, Bifidobacterium adolescentis	3,203 ± 4,211	2,266 ± 4,422	1993 ± 2,469	**0.0475**	0.8125	0.1094	2,765 ± 3,245	2,049 ± 3,977	0.4961	0.7998
Bacteria, Firmicutes, Clostridia, Ruminococcaceae, Blautia	1,020 ± 1,067	1,009 ± 1,312	66.25 ± 80.07	0.5209	**0.0156**	**0.0156**	557.6 ± 624.4	1,033 ± 1,406	> 0.9999	0.2389
Bacteria, Bacteroidetes, Bacteroidia, Bacteroidales, Bacteroidaceae, Bacteroides, bacterium NLAE-zl-H482	271.9 ± 1,102	148.9 ± 428.3	2.375 ± 4.069	0.9088	0.0625	**0.0156**	201.7 ± 485.1	174.0 ± 546.4	0.2031	0.7653)
Bacteria, Firmicutes, Clostridia, Ruminococcaceae Faecalibacterium unc.	445.5 ± 94.2	149.6 ± 357.2	0.3750 ± 1.061	0.0649	**0.0156**	**0.0313**	376.4 ± 602.1	285.9 ± 482.1	0.9999	0.4040
Bacteria, Actinobacteria, Coriobacteria, Coriobacteriaceae, Collinsella unc.	784.5 ± 708.5	740.8 ± 854.4	338.5 ± 273.4	0.7516	0.1563	**0.0156**	512.0 ± 303.9	593.1 ± 555.7	0.6953	0.3612
Bacteria, Firmicutes, Clostridiales, Ruminococcaceae, Ruminococcus 1 unc.	233.9 ± 599.1	211.2 ± 478.6	68.14 ± 64.28	0.8553	0.8125	0.8438	4.200 ± 9.852	18.80 ± 50.11	0.6445	0.1972
Bacteria, Firmicutes, Clostridiales, Ruminococcaceae, Fusicatenibacter	1,358 ± 1,251	1,572 ± 1,379	1985 ± 1,204	0.6091	0.4688)	0.5781	1,321 ± 653.0	1,653 ± 1,444	0.5566	0.5153
Bacteria, Firmicutes, Clostridia, Clostridiales, Ruminococcaceae, Subdoligranulum	232.7 ± 424.7	581.8 ± 1,033	905.6 ± 667.3	0.4818	**0.0313**	**0.0313**	413.4 ± 689.4	407.9 ± 687.6	0.9219	0.1025
Bacteria, Verrucomicrobia, Verrucomicrobia, Verrucomicrobiae, Verrucomicrobiales, Akkermansiaceae, Akkermansia	28.33 ± 159.2	11.91 ± 53.13	0.000 ± 0.000	0.9063	> 0.999	> 0.999	0.000 ± 0.000	0.2 ± 0.4216	0.5000	0.4498
Bacteria, Firmicutes, Clostridia, Clostridiales, Lachnospiraceae, Dorea, uncultured bacterium	1,117 ± 1,242	955.5 ± 1,523	1.125 ± 1.246	0.555	**0.0156**	**0.0156**	721.8 ± 771.8	421.0 ± 805.5	0.4258	0.8460

*The *p*-value evaluates whether the change in parameter values between T0–T6 is statistically significant. **The *p*-value evaluates whether the change in parameter values between T6–T12 is statistically significant. ***The *p*-value evaluates whether the change in parameter values between T0–T12 is statistically significant. ^†^The *p*-value evaluates whether the differences in parameter values between groups (SPs + vs. SPs−) are statistically significant at baseline (T0). The data are expressed as mean ± SD. Significant *p*-values are indicated in bold.

### Evaluation of Routine and Metabolic Parameters

A higher mean BMI value was observed in SPs+ patients than in SPs− patients (*p* = 0.0285) as well as glycemic values (*p* = 0.0001) at T0. The mean HOMA-IR value was found in SPs+ patients generally above normal values in the different study times (*p* > 0.05) defining this population as tendentially insulin resistant. In general, however, in all routine variables, no variation between T0–T6 and T6–T12 was found to be significant (*p* > 0.05, Table 1). An additional finding to report is the lack of change in both T0-T6 and T6-T12 liver transaminase values in both groups (i.e., AST and ALT, *p* > 0.05, see Table 1).

### Evaluation of Gastrointestinal Hormones and Peptides

In contrast to routine blood parameters, the gastrointestinal hormones and peptides we studied (i.e., aHSGF, GLP-1, PYY, and SeP) underwent profound changes during the evaluation time set for the study.

**FIGURE 2:**
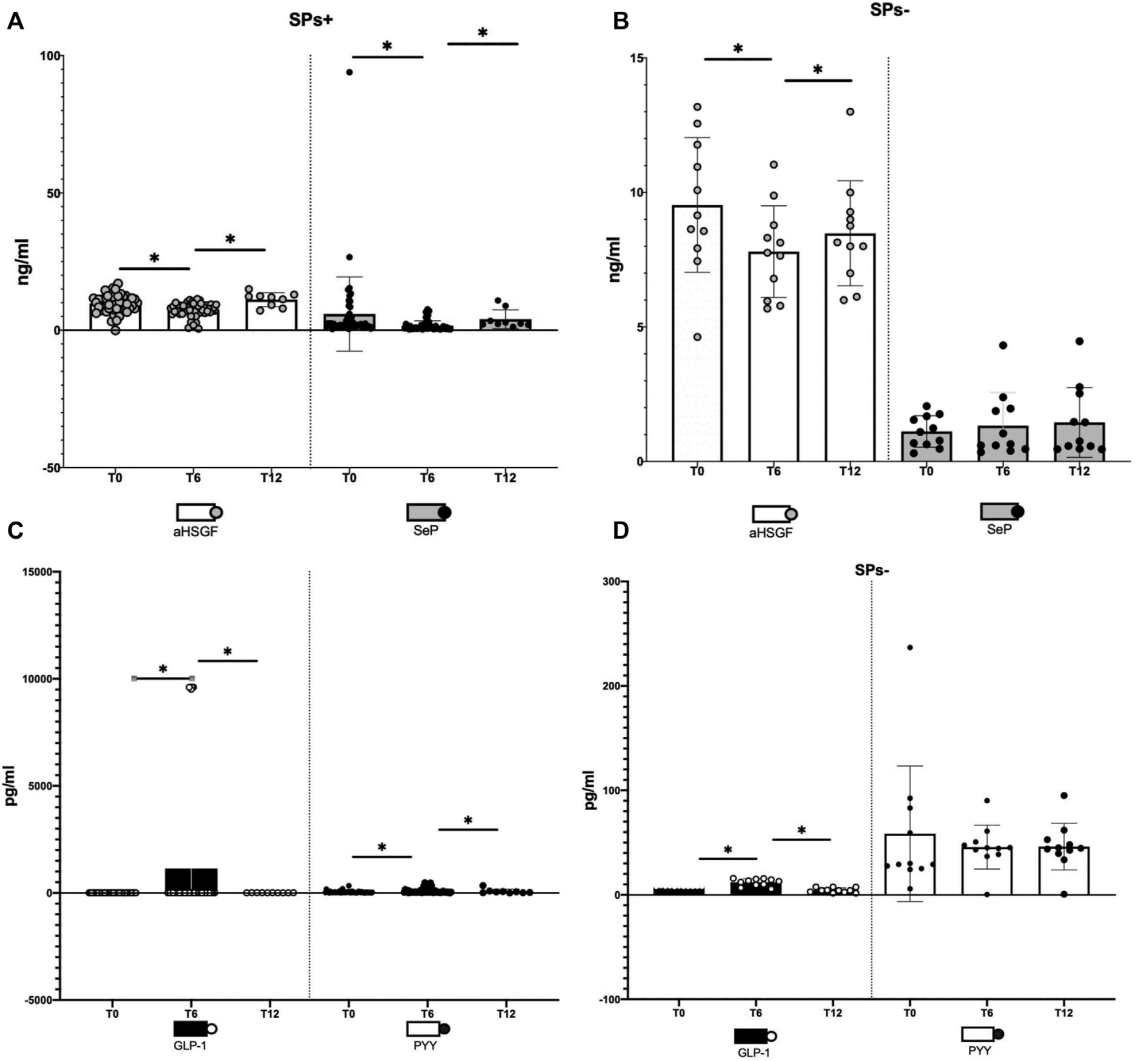
**(A–D)** Illustration of changes in aHSG-fetuin-A (aHSGF) and selenoprotein-P (SeP) levels in steatosis-positive, (SPs+) **(A)**, and steatosis-negative, (SPs−) **(B)**, patients. Illustration of changes in GLP-1 and YY peptide (PYY) levels in SPs+ **(C)** and SPs− **(D)** patients. Data are expressed as mean ± SD. ns: the variation is, in the ranges considered, not significant. *The variation is significant in the ranges considered.

At baseline (T0), GLP-1 levels were significantly higher in SPs− (*p* = 0.002) and SeP levels were lower in SPs− (*p* = 0.004). The levels of aHSGF were higher in SPs+, and those of PYY were lower in SPs+ (*p* > 0.05). However, this distribution confirmed the evidence available in the literature (Polyzos et al., 2020; Tarantino and Balsano, 2020). Starting from the aHSGF evaluation, a significant decreasing trend (*p* < 0.05, Table 1) was observed in both study groups (SPs+, SPs−). After 6 months of water intake (T0–T6), in both groups, a subsequent trend, equally significant, of restoration toward the initial values with reincrease of its value after wash-out (T6–T12) was observed. The same trend, in the SPs+, was accomplished by SeP, while in the SPs−, the trend was the opposite; however, changes in T0–T6 and T6–T12 times were significant exclusively for the SPs+ group (see Table 1). Another trend occurred for GLP-1 and PYY. In detail, GLP-1 has undergone an initial significant increase in the interval T0–T6 in both the SPs+ and SPs− populations, while it has undergone a subsequent significant decrease in values in the wash-out (T6–T12). The same behavior was true for PYY in the SPs+ population (see Table 1). Figures 2A,B summarize the trend for aHSGF and SeP, and Figures 2C,D show the trend for GLP-1 and PYY in SPs+ and SPs− populations, respectively.

In other words, condensing these results, the administration for 6 months of Fonte Essenziale® water has significantly ameliorated most of the blood concentrations of these hormones and peptides and removed the determining agent (wash-out period) these changes have moved, in large part significantly, in the opposite direction as if to restore the changes made in the 6 months of water intake. Moreover, another relevant observation is the fact that these changes were transversal, that is, not affecting only one group but both SPs+ and SPs− patients.

### Evaluation of the Oxidative Stress Parameters

As for the oxidation parameters examined at baseline (T0), none of them (i.e., TAC, TBARs, total thiols, and GSH) were significantly different between SPs+ and SPs−. Variations in the T0–T6 and T6–T12 intervals were, for the most part, non-significant, confirming the little impact of Fonte Essenziale® water on these variables (Table 1), except, however, for the increase in TAC in the T6–T12 interval, which was highly significant in SPs− (*p* = 0.001, Table 1).

### Assessment of the Gut Microbiota

At the start of the study, at T0, we had not observed a significant difference in the numerous bacterial species analyzed between the study groups (i.e., SPs+ and SPs−). Table 2 summarizes all changes in the bacterial species considered over the different study time intervals. Moving the assessment to T6, on the other hand, in the group of SPs+ patients, we observed a significant difference in Bifidobacterium adolescentis (Bacteria, Actinobacteria, Bifidobacteriaceae, Bifidobacterium adolescentis) (3,203 ± 4,211 vs. 2,266 ± 4,422, *p* = 0.0475), which decreased significantly over the 6 months. Other microbial populations did not show significant changes. The SPs− population, likewise, did not show significant changes in microbial composition in the T0–T6 period.

Finally, in a 12-month analysis, we observed several significant changes in the T0–T12 range in different microbial species in SPs+ patients:

• Bacteria, Firmicutes, Clostridia, Ruminococcaceae, and Blautia (1,020 ± 1,067 vs. 66.25 ± 80.07, *p* = 0.0156);
• Bacteria, Bacteroidetes, Bacteroidia, Bacteroidales, Bacteroidaceae, Bacteroides, and bacterium NLAE-zl-H482 (271.9 ± 1,102 vs. 2.375 ± 4.069, *p* = 0.0156);
• Bacteria, Firmicutes, Clostridia, and Ruminococcaceae Faecalibacterium unc. (445.5 ± 94.2 vs. 0.3750 ± 1.061, *p* = 0.0313);
• Bacteria, Actinobacteria, Coriobacteria, Coriobacteriaceae, and Collinsella unc. (784.5 ± 708.5 vs. 338.5 ± 273.4, *p* = 0.0156);
• Bacteria, Firmicutes, Clostridiales, Ruminococcaceae, and Subdoligranulum (232.7 ± 424.7 vs. 905.6 ± 667.3, *p* = 0.0313);
• Bacteria, Firmicutes, Clostridiales, Lachnospiraceae, Dorea, and uncultured bacterium (1,117 ± 1,242 avs. 1.125 ± 1.246, *p* = 0.0156).

In the same SPs+ patients, statistically significant differences in the T6–T12 range were observed for the following microbial populations:

• Bacteria, Firmicutes, Clostridia, Ruminococcaceae, and Blautia (1,009 ± 1,312 vs. 66.25 ± 80.07, *p* = 0.0156);
• Bacteria, Firmicutes, Clostridia, and Ruminococcaceae Faecalibacterium unc (149.6 ± 357.2 vs. 0.3750 ± 1.061, *p* = 0.0156);
• Bacteria, Firmicutes, Clostridiales, Ruminococcaceae, and Subdoligranulum (581.8 ± 1,033 vs. 905.6 ± 667.3, *p* = 0.0313);
• Bacteria, Firmicutes, Clostridiales, Lachnospiraceae, Dorea, and uncultured bacterium (955.5 ± 1,523 vs. 1.125 ±1.246, *p* = 0.0156).

**FIGURE 3:**
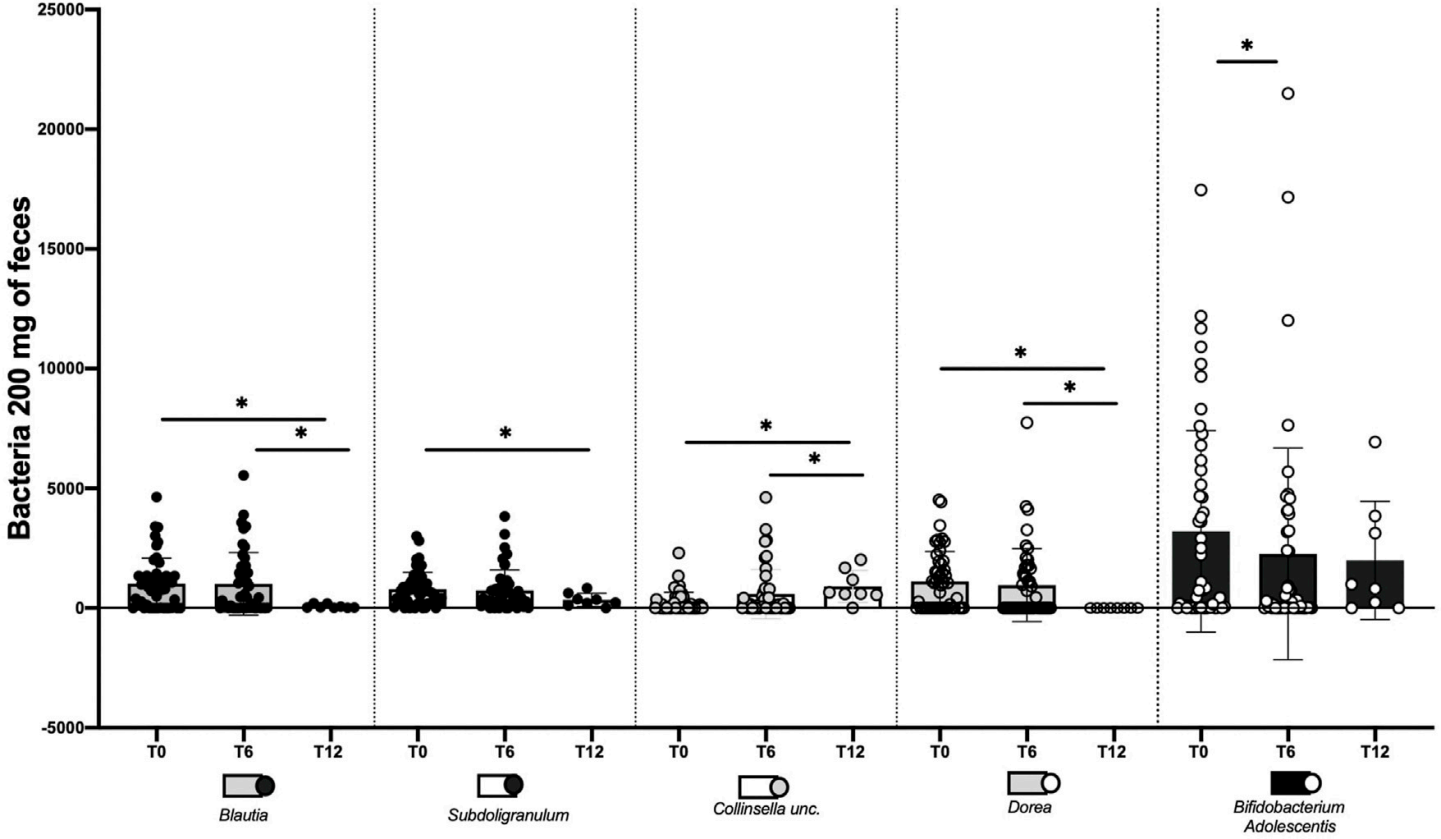
Changes made by water Fonte Essenziale ^®^ in the modification of some bacterial strains in steatosis-positive patients (SPs+). The assessment was performed at baseline (T0), after 6 months (T6), and after 12 months, i.e., after 6 months of water wash-out (T12). The data are expressed as mean ± SD. ns: the variation is, in the ranges considered, not significant. *The variation is significant in the ranges considered.

Differences in gut microbiota between the SPs− and SPs+ patient groups at T12 could not be identified as data for healthy patients are available for only few individuals. It was not possible to identify differences for the microbial species between the SPs− patient groups either at time T0–T12 or at time T6–T12; as already pointed out, only few individuals are available. The main changes in bacterial species in SPs+ patients are summarized in Figure 3.

We wanted, in addition, to query our data by selecting only SPs+ patients with elevated glycemia (>100 mg/dl) or with elevated ALT/γ-GT values, but we did not obtain results particularly different from those considering patients overall and not stratified concerning routine, oxidative, and gut microbiota parameters. The only exception was the reduction of ALT between T0–T6 in the SPs+ population with high ALT or γ-GT values (see Tables 3–6).

**TABLE 3:** Evaluation of study parameters assessed at baseline (T0), at 6 months (T6), and at 12 months (T12) in the special subset population of patients with steatosis (SPs+) and hyperglycemia (≥ 100 mg/dl).

Parameter	Steatosis-positive patients (SPs+) hyperglycemic				
	T0	T6	T12	*	**
BMI	30.01 ± 4.053	29.56 ± 3.973	29.75 ± 4.625	>0.999	>0.999
AST (UI/l)	22.22 ± 7.536	21.81 ± 5.477	33.00 ± 18.03	>0.999	0.07584
ALT (UI/l)	30.93 ± 18.77	24.63 ± 8.418	20.33 ± 6.658	0.8030	>0.999
γ-GT (U/l)	30.81 ± 19.54	27.56 ± 12.09	24.33 ± 8.327	0.7250	0.9111
Total cholesterol (mg/dl)	188.7 ± 38.65	192.5 ± 37.05	176.7 ± 30.09	0.8435	0.500
Triglycerides (mg/dI)	145.0 ± 58.83	161.1 ± 73.51	109.0 ± 50.51	0.5247	0.5391
Glycemia (mg/dl)	112.1 ± 12.49	111.9 ± 21.05	116.3 ± 12.58	0.9982	0.2500
Insulin (μU/ml)	15.41 ± 6.113	13.09 ± 7.100	13.41 ± 7.100	0.2140	0.2500
HOMA-IR	3.514 ± 1.728	3.776 ± 2.473	3.847 ± 1.151	0.8405	0.5000
TAC	0.2961 ± 0.076	0.3031 ± 0.0541	0.2613 ± 0.09534	0.8769	0.5000
TBARs (μM)	1.340 ± 0.4829	1.214 ± 0.4996	1.084 ± 0.5842	0.3581	0.5000
Total thiols (μM)	85.79 ± 65.58	100.8 ± 83.15	36.33 ± 13.19	0.6473	0.5000
GSH (mg/ml)	58.52 ± 51.50	76.77 ± 86.26	60.46 ± 47.47	0.3553	0.2500
aHSGF (ng/ml)	9.343 ± 3.080	7.120 ± 2.885	10.75 ± 2.443	**0.0012**	0.2500
GLP-1 (pg/ml)	4.315 ± 0.1740	1,072 ± 3,065	7.382 ± 2.020	**0.0008**	0.6250
PYY (pg/ml)	37.86 ± 37.55	96.00 ± 127.9	50.04 ± 22.43	**0.0113**	0.2500
SeP (ng/ml)	2.720 ± 3.080	1.495 ± 1.854	2.373 ± 0.4148	0.1430	0.5000

*The *p*-value evaluates whether the change in parameter values between T0–T6 is statistically significant. **The *p*-value evaluates whether the change in parameter values between T6–T12 is statistically significant. The data are expressed as mean ± SD. Significant *p*-values are indicated in bold.

**TABLE 4:** Evaluation of gut microbiota at baseline (T0), after 6 months (T6), and at 12 months (T12) in the special subset population of patients with steatosis (SPs+) and hyperglycemia (> 100 mg/dl).

Bacterial strain considered	Steatosis-positive patients (SPs+) hyperglycemic					
	T0	T6	T12	*	**	***
Bacteria, Actinobacteria, Bifidobacteriaceae, Bifidobacterium adolescentis	2,871 ± 3,383	2,209 ± 4,458	2,259 ± 2,893	0.6976	0.0293	0.0842
Bacteria, Firmicutes, Clostridia, Ruminococcaceae, Blautia	909.2 ± 965.3	1,129 ± 1,557	49.80 ± 77.18	0.6337	0.6291	0.1452
Bacteria, Bacteroidetes, Bacteroidia, Bacteroidales, Bacteroidaceae, Bacteroides, bacterium NLAE-zl-H482	456.3 ± 1,495	172.8 ± 394.8	3.000 ± 5.196	0.4159	0.0896	0.8242
Bacteria, Firmicutes, Clostridia, Ruminococcaceae Faecalibacterium unc.	455.6 ± 835.5	221.1 ± 471.2	0.6000 ± 1.342	0.4159	0.8242	0.0896
Bacteria, Actinobacteria, Coriobacteria, Coriobacteriaceae, Collinsella unc.	731.8 ± 533.5	713.0 ± 890.3	343.0 ± 284.6	0.9936	0.5747	0.3068
Bacteria, Firmicutes, Clostridiales, Ruminococcaceae, Ruminococcus 1 unc.	179.5 ± 610.4	192.9 ± 533.5	91.40 ± 61.49	0.9957	0.9784	0.8383
Bacteria, Firmicutes, Clostridiales, Ruminococcaceae, Fusicatenibacter	1,262 ± 1,063	1,422 ± 978.8	2,281 ± 1,236	0.8010	0.3690	0.3081
Bacteria, Firmicutes, Clostridia, Clostridiales, Ruminococcaceae, Subdoligranulum	288.2 ± 523.5	578.7 ± 908.8	710.8 ± 265.8	0.3892	0.8697	0.1551
Bacteria, Verrucomicrobia, Verrucomicrobia, Verrucomicrobiae, Verrucomicrobiales, Akkermansiaceae, Akkermansia	46.88 ± 216.4	8.560 ± 30.08	0.000 ± 0.000	0.6628	0.9310	0.2161
Bacteria, Firmicutes, Clostridia, Clostridiales, Lachnospiraceae, Dorea, uncultured bacterium	1,108 ± 1,420	822.8 ± 1,619	1.000 ± 1.414	0.5902	0.8441	0.4972

*The *p*-value evaluates whether the change in parameter values between T0–T6 is statistically significant. **The *p*-value evaluates whether the change in parameter values between T6–T12 is statistically significant. ***The *p*-value evaluates whether the change in parameter values between T0–T12 is statistically significant.

**TABLE 5:** Evaluation of study parameters assessed at baseline (T0), at 6 months (T6), and 12 months (T12) in the special subset population of patients with steatosis (SPs+) and elevated ALT or γ-GT levels.

Parameter	Steatosis-positive patients (SPs+) with elevated ALT or γ-GT Levels				
	T0	T6	T12	*	**
BMI	31.80 ± 4.665	31.07 ± 4.842	31.49 ± 8.321	0.5218	0.2500
AST (UI/l)	29.15 ± 6.930	25.30 ± 6.053	38.33 ± 12.74	0.1223	0.5000
ALT (UI/l)	41.30 ± 16.88	29.30 ± 6.689	29.67 ± 1.528	**0.0263**	>0.9999
γ-GT (U/l)	39.80 ± 39.45	31.10 ± 13.53	30.00 ± 17.35	0.6234	>0.9999
Total Cholesterol (mg/dl)	188.4 ± 35.16	197.9 ± 32.35	178.7 ± 20.40	0.5056	0.5000
Triglycerides (mg/dI)	127.2 ± 59.71	150.1 ± 69.78	81.67 ± 32.58	0.3727	0.2500
Glycemia (mg/dl)	103.0 ± 17.45	99.70 ± 16.51	94.00 ± 10.82	0.4139	0.2500
Insulin (μU/ml)	15.66 ± 7.496	12.39 ± 6.661	20.08 ± 12.99	0.1126	0.2500
HOMA-IR	3.536 ± 1.913	3.080 ± 1.712	4.350 ± 2.221	0.4636	>0.9999
TAC	0.2854 ± 0.0700	0.3055 ± 0.04108	0.1820 ± 0.1212	0.4710	0.2500
TBARs (μM)	1.270 ± 0.5065	1.199 ± 0.492	0.5940 ± 0.2757	0.8118	0.2500
Total thiols (μM)	91.79 ± 73.65	90.58 ± 80.79	59.55 ± 7.594	0.9981	0.7500
GSH (mg/ml)	62.96 ± 65.54	65.46 ± 67.69	34.94 ± 1.022	0.9859	0.5000
aHSGF (ng/ml)	10.62 ± 2.965	7.361 ± 2.837	12.77 ± 1.966	**0.0006**	0.2500
GLP-1 (pg/ml)	4.336 ± 0.1864	1,449 ± 3,517	7.566 ± 2.592	**0.0086**	0.6250
PYY (pg/ml)	32.19 ± 23.27	96.20 ± 125.9	42.78 ± 29.88	**0.0350**	0.2500
SeP (ng/ml)	4.710 ± 6.328	1.602 ± 1.871	4.887 ± 3.528	0.0846	0.5000

*The *p*-value evaluates whether the change in parameter values between T0–T6 is statistically significant. **The *p*-value evaluates whether the change in parameter values between T6–T12 is statistically significant. The data are expressed as mean ± SD. Significant *p*-values are indicated in bold.

**TABLE 6:** Evaluation of gut microbiota at baseline (T0), after 6 months (T6), and at 12 months (T12) in the special subset population of patients with steatosis (SPs+) with elevated ALT or γ-GT levels.

Bacterial strain considered	Steatosis-positive patients (SPs+) with elevated ALT or γ-GT levels					
	T0	T6	T12	*	**	***
Bacteria, Actinobacteria, Bifidobacteriaceae, Bifidobacterium adolescentis	3,700 ± 4,910	2,310 ± 4,765	1,641 ± 1744	0.4718	0.9569	0.402
Bacteria, Firmicutes, Clostridia, Ruminococcaceae, Blautia	1,129 ± 1,208	1,201 ± 1,323	86.00 ± 98.60	0.9750	0.0402	0.3357
Bacteria, Bacteroidetes, Bacteroidia, Bacteroidales, Bacteroidaceae, Bacteroides, bacterium NLAE-zl-H482	45.95 ± 74.47	101.1 ± 238.3	3.400 ± 4.980	0.5881	0.8341	0.0516
Bacteria, Firmicutes, Clostridia, Ruminococcaceae Faecalibacterium unc.	425.4 ± 931.4	174.5 ± 461.4	0.6000 ± 1.342	0.5881	0.8620	0.0636
Bacteria, Actinobacteria, Coriobacteria, Coriobacteriaceae, Collinsella unc.	750.3 ± 556.1	713.7 ± 893.3	327.5 ± 223.6	0.9850	0.4480	0.5790
Bacteria, Firmicutes, Clostridiales, Ruminococcaceae, Ruminococcus 1 unc.	138.3 ± 306.1	236.7 ± 627.1	69.60 ± 72.10	0.7505	0.9257	0.8767
Bacteria, Firmicutes, Clostridiales, Ruminococcaceae, Fusicatenibacter	1,393 ± 1,385	1,427 ± 1,342	2,276 ± 1,240	0.9885	0.4328	0.4714
Bacteria, Firmicutes, Clostridia, Clostridiales, Ruminococcaceae, Subdoligranulum	184.7 ± 336.5	447.6 ± 1,076	601.2 ± 418.7	0.5842	0.7817	0.1316
Bacteria, Verrucomicrobia, Verrucomicrobia, Verrucomicrobiae, Verrucomicrobiales, Akkermansiaceae, Akkermansia	64.11 ± 252.7	11.26 ± 34.27	0.000 ± 0.000	0.6450	0.8537	0.1221
Bacteria, Firmicutes, Clostridia, Clostridiales, Lachnospiraceae, Dorea, uncultured bacterium	1,354 ± 1,405	1,101 ± 1834	1.400 ±1.342	0.7656	0.6675	0.2535

*The *p*-value evaluates whether the change in parameter values between T0–T6 is statistically significant. **The *p*-value evaluates whether the change in parameter values between T6–T12 is statistically significant. ***The *p*-value evaluates whether the change in parameter values between T0–T12 is statistically significant.

## Discussion

In our study, we evaluated the effects of Fonte Essenziale® water concerning the gut–liver axis, metabolic parameters, oxidative stress parameters, and finally gut microbiota. Our study is the first study that considers this water as a medical device and applies it to evaluate these parameters in SPs+ and SPs−.

However, this result must be seen in the fact that water, although not an inert compound, as evaluated by our study, is not endowed with the pharmacodynamic properties typical of a canonical drug and therefore cannot have a direct effect on the pathogenic mechanisms of steatosis. In addition, since our population of SPs+ is diagnosed by ultrasound, it is assumed that the pathophysiological mechanisms have already had time to establish themselves decisively so that they cannot be drastically altered by the mineral water we evaluated.

As already explained, we also evaluated metabolic parameters. At baseline, we observed higher glycemia and BMI levels in SPs+ than in SPs−. At T6, we did not observe significant changes in BMI in SPs+, and this evaluation reinforces the fact that patients did not change their lifestyles while drinking water and that therefore, the beneficial effects do not directly depend on a change in BMI. At T6, we demonstrated a decrease in HOMA for insulin resistance (HOMA-IR) and insulinemia both in SPs+ and SPs−, although not statistically significant. BMI remains unchanged as well 6 months after the suspension of the Fonte Essenziale®, and this testifies, once again, that the patients, even in the period in which they did not drink the water, did not change their lifestyle. At T6–T12, we observed an increase in insulin value and HOMA-IR in both groups, although not significant.

Oxidative stress is known to play an established role in both pathogenesis and disease progression in non-alcoholic hepatic steatosis (Paradies et al., 2014; Cobbina and Akhlaghi, 2017; Dallio et al., 2021). So much so that subjects on a high antioxidant diet were found to have a preventive advantage over NAFLD in two case-control studies (Salehi-Sahlabadi et al., 2020; Sohouli et al., 2020). A diet with a strong antioxidant impact also showed a protective role in the histological severity of the disease. In particular, it seems that subjects with a dietary intake with a high dietary total antioxidant capacity would show a reduced presence of hepatocyte ballooning (De Oliveira et al., 2019). Low levels of total thiol levels have also been reported in patients with NAFLD (Tavakoli et al., 2020). Despite the lack of significance, we observed the results in line with evidence of total thiol and higher GSH in SPs− at the T0 assessment. Six months after water consumption (T6), none of the four evaluated oxidative stress parameters changed significantly. However, we observed a GSH increase in SPs+ while, on the contrary, a decrease TBARs. Even in the healthy population, the changes were not significant; however, an increase in GSH was interesting. At T12, we did not demonstrate a statistically significant variation of evaluated oxidative stress parameters concerning T6.

Several hormones have been included in the pathophysiological pathway of NAFLD, such as GLP-1, PYY, SeP, and aHSGF. At T0, GLP-1 and PYY levels appear to be quantitatively reduced in patients with NAFLD, whereas SeP and aHSGF appear to be increased, according to the actual evidence (D’Alessio, 2016; Petta et al., 2016; Bifari et al., 2018). At T6, we demonstrated an increase of GLP-1 and PYY compared to T0 and a decrease of SeP and aHSGF in SPs+. These results, taken together, confirm one of the outcomes of our study, showing that Fonte Essenziale® water has a beneficial effect in patients with hepatic steatosis for gastrointestinal hormones and peptides that we studied with an overall improvement at 6 months after use. We believe that the change in GLP-1 levels is worthy of attention. In fact, due to its effects of stimulating insulin production, inhibiting glucagon, and reducing appetite at the central level, it plays a role in the context of insulin resistance, which has already been included several times in the pathogenesis of NAFLD and as a basis for the development of insulin resistance (Watt et al., 2019). The same observations may be related to SeP, which has also shown a direct metabolic link to NAFLD, increasing circulating LDL levels and facilitating the development of insulin resistance (Petta et al., 2016; Kitade et al., 2017).

Another important aspect we evaluated in our study is the gut microbiota. Several microbial populations have shown interesting behavior over the assessment times. Blautia showed a reduction in T6 in SPs + patients (*p* = 0.5209). This trend is confirmed as well at 12 months by comparing both T0–12 and T6–T12 with a significant reduction at 12 months (*p* = 0.0156).

Shen et al. (2017) have shown that in patients with NASH, there is a greater representation of the genus Blautia, suggesting a decisive role in the progression of NAFLD. Our study showed a reduction of this bacterial type with a total beneficial cumulative effect.

Collinsella unc. in SPs+ patients at T6 showed a non-significant decreasing trend compared to T0 (*p* = 0.7516). This decreasing trend was confirmed at T12 both by comparison of T0–T12 (*p* = 0.0156) and by comparison of T6–T12 (*p* = 0.1563). In other words, this bacterium’s behavior was like that of Blautia. In a recent study published on gut microbes (Astbury et al., 2020), authors characterized gut microbiota composition in United Kingdom patients with biopsy-proven NASH and compared it to that in healthy controls. The genus strongly associated with NASH in this study was Collinsella. This genus, which has been linked previously to obesity and atherosclerosis, was also positively correlated with fasting levels of triglycerides and total cholesterol and negatively correlated with high-density lipoprotein cholesterol, suggesting that some of the pathways present in this microbial genus may influence the lipid metabolism in the host. Fonte Essenziale® water has thus led to a reduction of a bacterial species potentially associated with the onset and progression of NAFLD. In addition, an increase in Subdoligranulum was observed at T6 (*p* = 0.4818) in SPs+ patients.

The upward trend is confirmed at T12 both by comparison of T0–T12 (*p* = 0.0313) and by comparison of T6–T12 (*p* = 0.0313), with the increase being statistically significant after 12 months. Van Hul et al. (2020) revealed, by analyzing both qPCR and shotgun metagenomic data, that the abundance of Subdoligranulum was correlated positively with microbial richness and HDL-cholesterol levels and negatively correlated with fat mass, adipocyte diameter, insulin resistance, levels of leptin, insulin, CRP, and IL-6 in humans. We then observed the growth of a bacterial species potentially associated with “good” factors (metabolic, inflammatory, and others) preventing the onset and worsening of NAFLD.

Dorea is another microbial species that showed a non-significant reduction trend at 6 months (*p* = 0.555) in the T0–T6 comparison with significant confirmation even at 12 months both between T0–T12 (*p* = 0.0156) and between T6–T12 (*p* = 0.0156). Del Chierico et al. (2017) have shown how NAFLD patients had increased levels of Bradyrhizobium, Anaerococcus, Peptoniphilus, Propionibacterium acnes, and Dorea and reduced proportions of Oscillospira and Rikenellaceae compared to controls. After reducing metagenomics and metabolomics data dimensionality, multivariate analyses indicated a decrease in Oscillospira in NAFLD and NASH groups and increases of Blautia and Dorea in NASH patients compared to controls. Therefore, an increase in Dorea was identified as a gut microbiota signature of NAFLD onset and NAFLD-NASH progression. Again, our study showed that the intake of the water under assessment results in a reduction of a bacterial species potentially associated with the onset and progression of NAFLD. In addition, a decrease in Faecalibacterium unc. was observed at T6 (*p* = 0.0649).

The decreasing trend is confirmed at T12 both by comparison of T0–T12 (*p* = 0.0313) compared with T6–T12 (*p* = 0.0156), with a statistically significant reduction after 12 months, although there is still too little evidence in the literature on this bacterial species and its role in NAFLD. For the moment, it is known that it is an anti-inflammatory commensal stimulating the secretion of IL-10 and inhibiting the expression of IL-12 and interferon-γ and that it is reduced in many of the NAFLD patients, reduced due to the “NAFLD-signature,” which is a particular profile representing the various intestinal microbial species, characterized by an increase in the levels of bacteria and a reduction in the levels of bacteria, typical of NAFLD patients and non-healthy ones (Grabherr et al., 2019).

Bifidobacterium adolescentis showed a significant reduction trend in the T0–T6 range (*p* = 0.0475), while despite the fact that the confirmation of a decrease trend was also observed at T12, comparing T0–T12 (*p* = 0.1094) and T6–T12 (*p* = 0.8125), the trend was not significant.

Several strains of Bifidobacterium adolescentis have been associated with a reduction in the expression of pro-inflammatory mediators such as IL-1β, TNF-α, and NF-κBp65; induction of T-helper 17 cells in humans and rodents; a significant reduction in ROS formation; regulation of the immune system; and driving the worsening of various conditions, including NAFLD-NASH progression, as supported by various pieces of evidence (Guo et al., 2019; Yu et al., 2019). Some strains of B. adolescentis relieve NAFLD by varying the concentration of certain short-chain fatty acids (SCFAs), including butyrate, in the intestine of NAFLD mice (Wang et al., 2020).

SCFAs activate GPR41 and GPR43 receptors on L-enteroendocrine cells by stimulating the secretion of PYY and GLP-1 and activating the AMPk kinase pathway, which reduces oxidative stress and inflammation and activates T reg cells (Chu et al., 2019). Murine models fed a “Western-style” diet without concomitant administration of probiotics containing B. Adolescentis gained weight and developed steatohepatitis in contrast to murine models fed the same diet but with concomitant B. adolescentis, which showed significantly reduced liver damage (Reichold et al., 2014). In addition, in Wistar rats, integration of B. adolescentis enhances visceral fat accumulation and insulin sensitivity in an experimental pattern of metabolic syndrome (Chen et al., 2012). Some strains of B. adolescentis have been associated with increased expression of brain-derived neurotrophic factor in the hippocampus (Guo et al., 2019), suggesting, in addition to the above, also a potential anxiolytic and antidepressant role. A reduction in Bifidobacteriaceae was demonstrated in a recent study (Ponziani et al., 2019) to investigate which characteristics of the gut microbiota were associated with HCC in patients with NAFLD-related cirrhosis.

Overall, although in the study of GLP-1, PYY, aHSGF, and SeP we observed a sort of “restoration” of their values in the wash-out compared with T0, the levels of several bacterial species of the gut microbiota at T12 remained substantially different from those at baseline (Table 2), indicating a more pronounced effect of water in the microbiota than enterokines after wash-out. Daily dietary intake provides carbohydrates that are not digested and therefore provide fuel for bacterial growth. These carbohydrates are metabolized by gut bacteria in the complex interaction between the host and gut microbiota (Albillos et al., 2020). These carbohydrates are metabolized by gut bacteria into SCFAs in that complex interaction between the host and gut microbiota. It has already been observed that providing SCFAs improves high-fat diet-induced hepatic steatosis in mice (Albillos et al., 2020). However, one of the limitations of the present study was the failure to assay SCFA levels, and new studies will probably better clarify this question.

All the above considerations lead us to conclude that Fonte Essenziale® water was not an inert compound because it significantly modified several gastrointestinal hormones and gut bacterial flora. However, there was no drastic reduction in liver steatosis. This fact, according to us, is explainable because first, as already said, the diagnosis was only done by ultrasound and second, the administration was practiced only for 6 months. As already explained, at T12, most of these effects are lost. Certainly new, larger, double-blind controlled studies, with a longer follow-up, will be able to better clarify the short- and long-term effects of Fonte Essenziale^®^ water in the perspective of a non-pharmacological therapy of NAFLD.

## Data Availability

The data presented in the study are deposited in the European Nucleotide Archive (ENA) repository, accession number PRJEB52995.
